# Case Report: Zero-contrast balloon pulmonary angioplasty guided by real-time fusion of CT angiography with x-ray fluoroscopy

**DOI:** 10.3389/fcvm.2024.1435795

**Published:** 2024-11-18

**Authors:** Yinjiang Tang, Ou Xu, Chunmei Zhang, Xia Bao, Shaodong Ye

**Affiliations:** ^1^ Department of Pulmonary Vascular and General Medicine, Fuwai Yunnan Cardiovascular Hospital, Yunnan Provincial Cardiovascular Disease Clinical Medical Center/Affiliated Cardiovascular Hospital of Kunming Medical University, Kunming, Yunnan, China; ^2^ Department of Cardiology, Fuwai Hospital, Chinese Academy of Medical Sciences, Beijing, China

**Keywords:** real-time image fusion navigation technique, chronic thromboembolic pulmonary hypertension, balloon pulmonary angioplasty, pulmonary endarterectomy, pulmonary artery

## Abstract

**Background:**

Allergy to iodine contrast agents has long been a contraindication for balloon pulmonary angioplasty (BPA). We report the successful zero-contrast BPA procedure of a patient with inoperable chronic thromboembolic pulmonary hypertension (CTEPH) and severe iodine allergy using real-time fusion of computed tomography angiography (CTA) with x-ray fluoroscopy.

**Case presentation:**

A 62-year-old woman with CTEPH who developed a severe allergic reaction after the first BPA procedure. The second BPA procedure was successfully performed using the fusion of previous pulmonary artery CTA images and real-time fluoroscopy images. Pulmonary artery pressure showed a significant decrease after performing BPA twice. As of 6-month follow-up, the patient has recovered well.

**Conclusion:**

3 Dimensions (3D) reconstruction of CTA images and real-time fluoroscopic image fusion navigation technology provide a new option for BPA treatment in patients allergic to iodine contrast agents.

## Introduction

Chronic thromboembolic pulmonary hypertension (CTEPH) is a potentially life-threatening condition, characterized by obstruction of pulmonary artery by chronic thrombosis. The pathophysiology of CTEPH involves pulmonary vascular remodeling and luminal stenosis or occlusion, leading to a progressive increase in pulmonary artery pressure. Untreated CTEPH may lead to right heart failure (1, 2). Pulmonary endarterectomy (PEA) is the gold standard treatment for CTEPH. However, the thromboembolic lesions below the pulmonary artery segment may not be amenable to PEA. Balloon pulmonary angioplasty (BPA) is the current consensus treatment for patients not suitable for PEA (3, 4). Standard protocols of BPA use iodine-based contrast agents (5). Therefore, BPA is contraindicated in CTEPH patients who are severely allergic to iodinated contrast agents (6, 7). In this paper, we report a case of CTEPH with severe hypersensitivity to iodinated contrast agents. Three-dimensional reconstruction of the patient's pulmonary artery CTA image and real-time image fusion navigation were used to perform BPA without the use of a contrast agent.

## Case presentation

A 62-year-old woman was hospital admitted with the diagnosis of acute pulmonary embolism (intermediate risk). She was prescribed oral long-term anticoagulant therapy with rivaroxaban. Four months later, the patient was admitted to the hospital for a second review, and clinical symptoms of chest tightness and dyspnea during activity were still persistent. The computed tomography pulmonary angiogram (CTPA) showed that the patient presented remain pulmonary embolism. The mean pulmonary artery pressure (mPAP) was 33 mmHg, mean pulmonary artery wedge pressure (mPAWP) was 12 mmHg, cardiac output value (CO) was 4.75 L/min and the pulmonary vascular resistance (PVR) was 4.63 Wood units. Using a noncompliant ballon (NC sprinter, Medtronic), the BPA was performed at the following segmental arteries: anterior branch of right superior pulmonary artery (RA3), medial branch of right basal pulmonary artery (RA7), lateral branch of right basal pulmonary artery (RA9) and posterior branch of right basal pulmonary artery (RA10b). The amount of iodine contrast medium used during BPA was 280 ml. On the second day after percutaneous procedure, the patient developed facial edema, skin itching and rash all over the body, pharyngeal spasms with mild dyspnea, which was diagnosed as having an allergic reaction to the iodine contrast agent. The symptoms were relieved after 2 days of anti-allergic drug treatment. And then the patient begins to receive the treatment with Riociguat.

**Figure 1:**
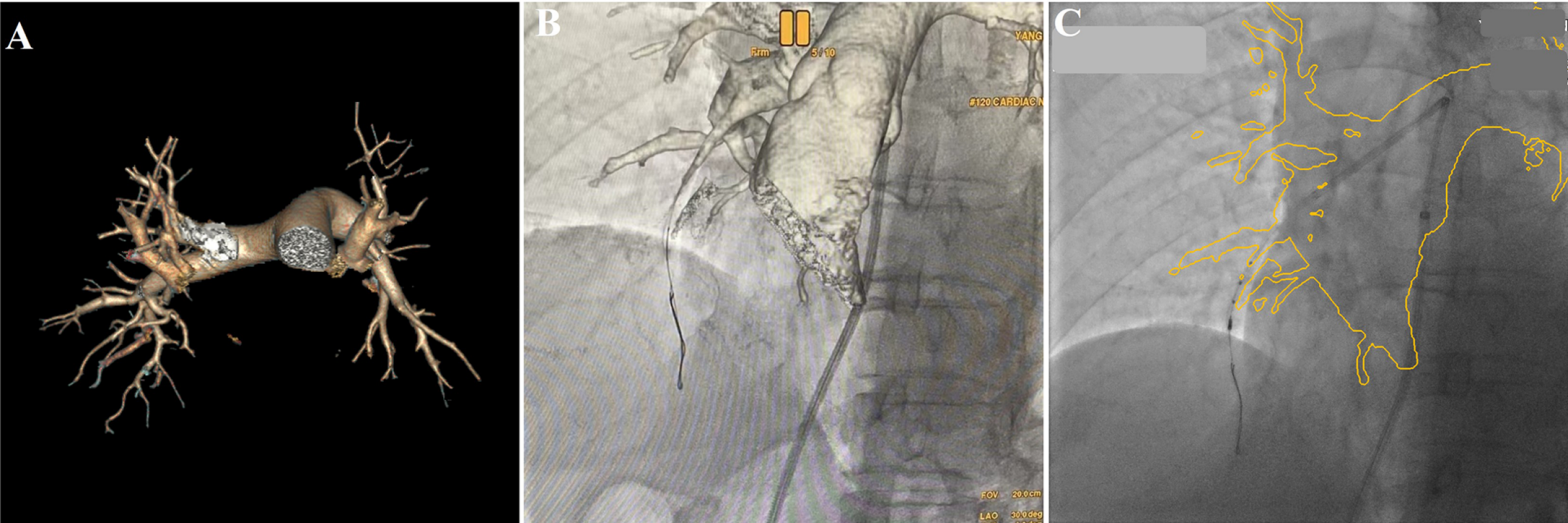
Virtual reality reconstruction of the patient's pulmonary artery based on previous pulmonary artery CTA image **(A)**, real-time image fusion navigation guided the direction of the guidewire via pulmonary artery to A7 (**B**, Virtual reality), and guided the IUVS into A8a (**C**, outline volume-rendered) during BPA procedure.

Six months later, the patient was re-admitted for re-examination, and right heart catheterization was performed which showed mPAP of 28 mmHg, mPAWP of 8 mmHg, CO of 5.1 L/min and PVR of 3.92 Wood units. As the use of an iodine contrast agent was contraindicated, BPA procedure was performed by the following methods: the patient's previous multidetector computed tomography images were transmitted back to the AW4.7 workstation (GE Healthcare), and the AutoBoneXpress function was selected to manually extract a part of trachea and spine respectively, and improve the following registration precision and speed. At the same time, virtual reality reconstruction of the pulmonary artery vascular structure was performed and marked with the image fusion software Value Assist 2; the spine and trachea were registered, and the view switched to the vascular image to start intraoperative real-time navigation (IGS530, GE Healthcare) for performing BPA (Figure 1). It can guide the direction of the guidewire within the pulmonary artery without contrast agent assisted tracing, ensuring more efficient navigation through narrowed areas of the pulmonary artery. Additionally, it also provides real-time guidance on the positioning of the guidewire and balloon during interventional procedures.

**Figure 2:**
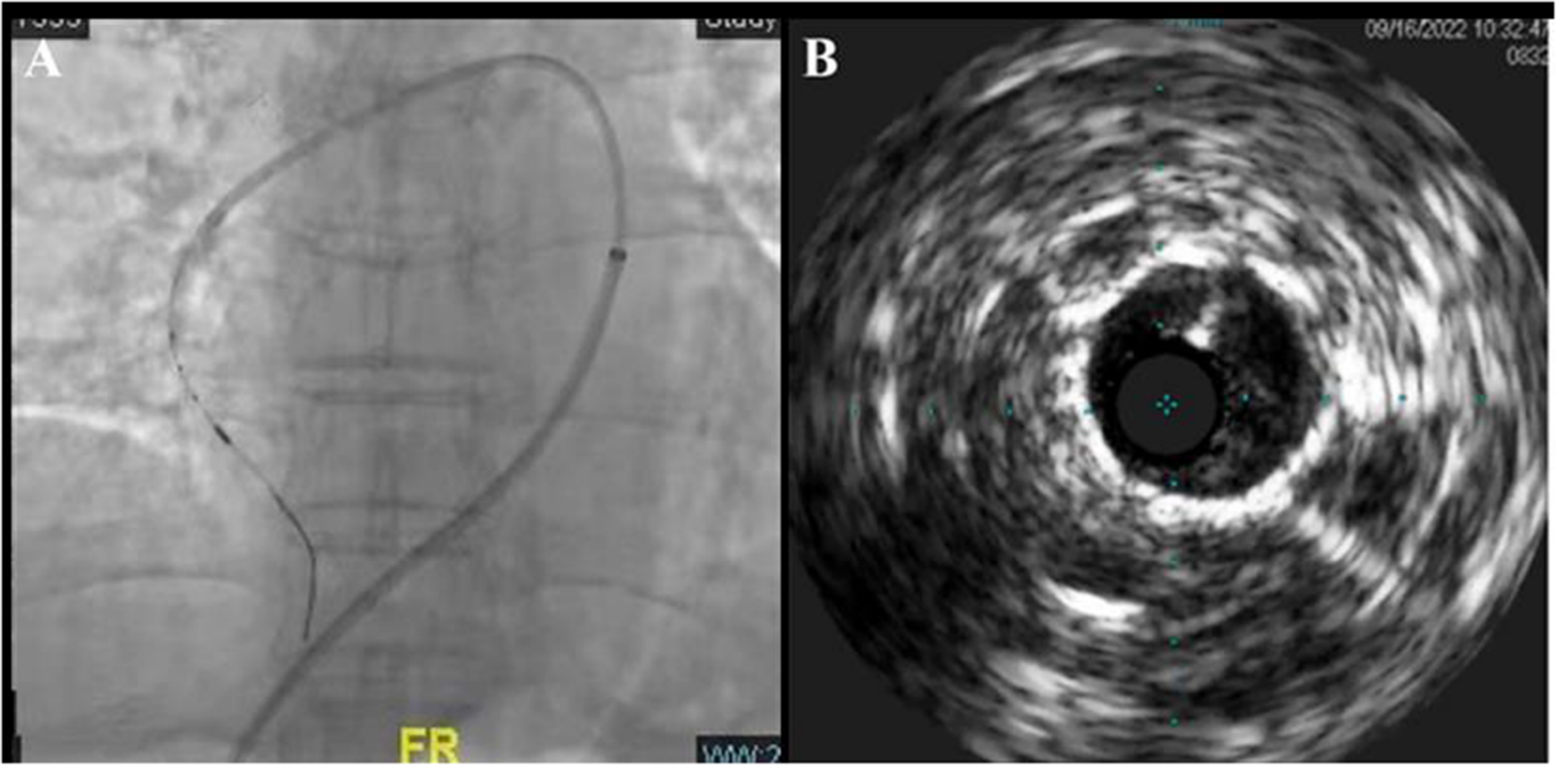
Real-time image fusion navigation technique and IVUS-guided BPA **(A)** and its intraluminal structure **(B)**.

The pressure guide wires were placed into RA3, RA7, RA9, and RA10b, respectively, under real-time fusion of x-ray fluoroscopy and CTA 3D reconstruction images. The severity of a lesion in the pulmonary artery was evaluated by difference in pressure gradient between the proximal and distal sides of the lesion. The Pd/Pp ratio (the ratio of mean distal to mean proximal pressures) of all the above-mentioned blood vessels was greater than 0.7. At the entry of the pressure guidewire (Certus™, Abbott Medical) into the lateral branch of the right basal pulmonary artery (RA8a), the Pd/Pp ratio in the middle part of RA8a was the lowest at 0.48. Intravascular ultrasound (IVUS) was performed for the middle part of RA8a through angiographic navigation (Figure 2), and the diameter and length of the stenosis were measured. The balloon (Sprinter 3.0 × 15 mm) was selected according to the lumen diameter and placed into the stenosis of the middle RA8a, and segmental dilatation plasty (6–16 atm) was performed. After the plasty, the Pd/Pp ratio of the middle RA8a was 0.78, as measured by the pressure guide wire. Immediately after percutaneous procedure, which showed mPAP of 24 mmHg, mPAWP of 7 mmHg, CO of 5.4 L/min and PVR of 2.98 Wood units.

No contrast agent was used in the entire 90 min operation, and the patient experienced no discomfort. During the 6-month follow-up after the operation, the patient showed clinical improvement and had no chest tightness or dyspnea while performing daily activities.

## Discussion and conclusion

BPA is an important method for pulmonary artery revascularization, especially revascularization below the pulmonary artery segment. A successful BPA procedure depends on the use of iodized contrast media and two-dimensional digital subtraction angiography. According to the WHO, iodine contrast agents are used by more than 75 million people worldwide every year, and these agents are well tolerated by most patients. However, some patients develop serious and potentially fatal adverse reactions (8, 9). Szymon and colleagues (10) reported on 250 BPA procedures for 41 patients with CTEPH from 2013 to 2017. One patient had a severe contrast allergy reaction, and two developed CI-AKI, but none needed dialysis. The contrast used was within safe limits based on their kidney function. After three months, the kidney function of those with CI-AKI returned to normal. The study indicates that while contrast allergies are hard to avoid in BPA, they can be severe. Therefore, the ability to perform BPA without the use of a contrast agent portends good for these patients.

Image fusion navigation is based on CTA and MRA data reconstruction and accurate registration. The 3D blood vessel image and real-time fluoroscopy fusion are superimposed on the fluoroscopy screen and the C-arm movement is followed to achieve real-time intraoperative guidance. A previous study has reported that 3D image fusion technology can help significantly reduce the amount of contrast agents (11, 12). In the present case, preoperative CTA data and real-time fluoroscopy were used to guide BPA treatment. A pressure guide wire was used during the operation to determine the Pd/Pp ratio of each pulmonary artery vessel to identify the lesion vessels and avoid complications such as pulmonary edema. In addition, IVUS was used to determine the degree of stenosis of the lesion site to guide BPA. As of 6-month follow-up, the patient has shown clinical improvement. Pulmonary artery pressure showed a significant decrease after performing BPA twice, and no contrast agent was used during the second BPA treatment. It also suggests that BPA without contrast, using real-time fusion of CT angiography with x-ray fluoroscopy, is a feasible option for CTEPH patients with contrast agent allergies or renal insufficiency. To the best of our knowledge, the use of 3D image fusion technology for BPA has not been reported in domestic and foreign literature, and this technology is worthy of further exploration and development.

In this case, we took a reliable measure to prevent the use of iodine contrast agent in second BPA procedure. Its success hinged on preoperative CTA, which did use iodine contrast. Additionally, it may still be necessary to utilize contrast agents for ensuring the efficacy and safety in the treatment of complex lesions, including those involving tortuous and occlusive lumens within the pulmonary vasculature, as well as to manage intraoperative complications, such as pulmonary hemorrhage. The application of real-time fusion navigation technology is a proven method to minimize iodine contrast agent usage. In summary, we report the successful zero-contrast BPA management of a patient with inoperable CTEPH and severe iodine allergy using real-time fusion of CT angiography with x-ray fluoroscopy. Real-time fusion navigation may be an attractive treatment option for patients with inoperable CTEPH and severe iodine allergy.

## Data Availability

The original contributions presented in the study are included in the article/Supplementary Material, further inquiries can be directed to the corresponding author.
